# Localized large language model TCNNet 9B for Taiwanese networking and cybersecurity

**DOI:** 10.1038/s41598-025-90320-9

**Published:** 2025-03-20

**Authors:** Jiun-Yi Yang, Chia-Chun Wu

**Affiliations:** 1 Department of AI & Data, Hi5 Technology Incorporation, Taipei, Taiwan; 2 DataAgent Incorporation, Taipei, Taiwan; 3 Department of Computer Science and Information Engineering, National Quemoy University, Kinmen, Taiwan

**Keywords:** Large language model (LLM), Domain adaptation, Networking application, Cybersecurity, Finetuning, Computer science, Information technology

## Abstract

This paper introduces TCNNet-9B, a specialized Traditional Chinese language model developed to address the specific requirements of the Taiwanese networking industry. Built upon the open-source Yi-1.5-9B architecture, TCNNet-9B underwent extensive pretraining and instruction finetuning utilizing a meticulously curated dataset derived from multi-source web crawling. The training data encompasses comprehensive networking knowledge, DIY assembly guides, equipment recommendations, and localized cybersecurity regulations. Our rigorous evaluation through custom-designed benchmarks assessed the model’s performance across English, Traditional Chinese, and Simplified Chinese contexts. The comparative analysis demonstrated TCNNet-9B’s superior performance over the baseline model, achieving a 2.35-fold improvement in Q&A task accuracy, a 37.6% increase in domain expertise comprehension, and a 29.5% enhancement in product recommendation relevance. The practical efficacy of TCNNet-9B was further validated through its successful integration into Hi5’s intelligent sales advisor system. This research highlights the significance of domain-specific adaptation and localization in enhancing large language models, providing a valuable practical reference for future developments in non-English contexts and vertical specialized fields.

## Introduction

The rapid advancement of large language models (LLMs) has revolutionized natural language processing (NLP), enabling the creation of sophisticated AI applications with high accuracy. Despite these advancements, there remains a gap in the localization and specialization of these models for specific domains and languages. This paper addresses this gap by introducing TCNNet-9B, a Traditional Chinese language model tailored for the networking domain, built upon the open-source Yi-1.5-9B model. Through continued pretraining and finetuning, TCNNet-9B has been developed to meet the unique needs of the Taiwanese networking industry.

Current third-party models such as Open AI’s GPT-4 and Google’s Gemini possess substantial knowledge of international networking concepts but lack a comprehensive understanding of local brands, regulations, and consumer preferences unique to Taiwan. This limitation underscores the need for a domain-specific, culturally aware model that can provide accurate and relevant information in Traditional Chinese. TCNNet-9B aims to fill this gap by leveraging localized datasets and advanced finetuning techniques, thereby retaining the capabilities of its base model while excelling in providing detailed, localized knowledge and practical recommendations for the Taiwanese networking industry.

The research collected a rich corpus of Traditional Chinese content through web scraping, including articles on networking knowledge, DIY assembly guides, equipment recommendations, and cybersecurity information pertinent to Taiwan. Additionally, local cybersecurity regulations, industry trends, and brand information were incorporated to enhance the model’s contextual understanding. Finetuning the model for the Q&A and product recommendation tasks ensured its ability to handle specific user queries and provide relevant suggestions. TCNNet-9B’s performance, benchmarked against other models using the Taiwanese Networking and Cybersecurity Knowledge Benchmark (TNCK-Bench), demonstrates its superior ability to provide domain-specific and culturally relevant responses, making it a valuable tool for the Taiwanese networking industry.

## Literature review

### Introduction to large Language models (LLMs)

Large language models (LLMs) have fundamentally transformed natural language processing (NLP), enabling the development of sophisticated AI applications that understand and generate human language with remarkable accuracy. Foundational models like GPT-4 and Gemini-1.5 have demonstrated significant advancements across various tasks, including text completion, translation, and summarization, leveraging vast datasets and billions of parameters to capture linguistic patterns and contextual nuances^1,2^. These advancements set the stage for specialized models like TCNNet-9B, which cater to Taiwan’s specific linguistic and cultural needs.

### Domain-specific adaptation and importance of knowledge in LLMs

The adaptation of large language models (LLMs) for specific domains has become a focal point in recent research, emphasizing the necessity of integrating domain-specific knowledge to enhance model performance. General-purpose models, such as GPT-4 and Gemini-1.5, have demonstrated remarkable capabilities in various tasks; however, they often fall short when applied to niche applications due to their lack of familiarity with specialized terminology and contextual nuances inherent to those fields.

Research indicates that finetuning LLMs on specialized datasets significantly improves their relevance and accuracy in targeted applications. For instance, studies have shown that models trained with domain-specific content exhibit enhanced understanding and generation capabilities, particularly in fields such as healthcare and finance^3–5^. Particularly pertinent to this discussion is TCNNet-9B, a model explicitly developed for the Taiwanese networking industry. This specialized model leverages a meticulously curated dataset, encompassing local cybersecurity regulations, comprehensive networking knowledge, and industry-specific equipment recommendations. By incorporating these domain-specific elements, TCNNet-9B exemplifies the potential of targeted language models in addressing a specific sector’s unique challenges and requirements within a localized context^6^.

Incorporating domain-specific knowledge not only aids in vocabulary acquisition but also enhances the model’s ability to understand user intent and contextual factors influencing decision-making within a particular field. For example, the framework proposed by Yao et al. (2023) introduces a paradigm that augments LLMs with domain-specific knowledge through a systematic approach, demonstrating how such enhancements can to improved performance on practical applications like recommender systems^7^. Similarly, Gawade et al. (2023) present a novel integration method that infuses relevant knowledge into LLMs, significantly boosting their domain-specific understanding while reducing computational costs^8^.

Furthermore, the effectiveness of domain adaptation is closely tied to the quality and relevance of the training data. By embedding localized datasets that reflect the unique characteristics of a specific industry or region, researchers can create models that deliver actionable insights tailored to the needs of professionals in that domain. Zhang et al. (2024) propose a framework for incorporating knowledge bases into LLMs for domain-specific question answering, emphasizing the importance of aligning model responses with user preferences^9^. This approach highlights how effective integration of domain knowledge can enhance user satisfaction and model applicability.

Moreover, recent work by Lu, R.-S. et al. (2024) explores retrieval-augmented generation techniques that empower LLMs with domain-specific knowledge in educational contexts^10^. Their findings further support the notion that leveraging specialized information can significantly improve model outputs across various applications.

In summary, incorporating domain-specific knowledge is essential for optimizing LLM performance in specialized applications. This necessity underlines the approach taken in developing TCNNet-9B, which aims to bridge the gap between general language processing capabilities and the specific requirements of Taiwan’s networking sector.

### Localization of LLMs for Non-english languages

The performance of large language models (LLMs) in non-English languages has become a critical area of study, particularly as the demand for localized AI applications grows. While multilingual models such as LLaMA, Qwen, Yi, and Mistral have demonstrated capabilities across various languages, their effectiveness can be significantly enhanced through targeted localization efforts. Localization involves translating text and adapting models to understand cultural nuances, idiomatic expressions, and specific regional contexts that influence language use.

Research indicates that LLMs trained on multilingual datasets often struggle with languages that possess unique syntactic and semantic structures. Traditional Chinese, for example, presents unique linguistic challenges due to its complex grammatical structures, intricate writing system, and culturally specific references. These distinctive features are often inadequately represented in models primarily trained in English or other Western languages, leading to potential misinterpretations or inaccuracies in language processing tasks. The logographic nature of Chinese characters, tonal system, and context-dependent meanings further compound the difficulties in achieving accurate language understanding and generation for AI models not specifically tailored to handle these linguistic intricacies. Studies by Adilazuarda et al. (2024) highlight how localization efforts can improve model performance by ensuring that the training data reflects the linguistic characteristics and cultural contexts of the target language^11^.

One effective strategy for localization is to finetune LLMs on large datasets curated explicitly for the target language. This approach helps models learn not only vocabulary but also the contextual usage of terms within specific domains. For example, Lin and Chen (2023) demonstrated that models trained on localized datasets exhibit improved comprehension and generation capabilities in Traditional Chinese, particularly in technical fields like networking and cybersecurity^12^.

In addition to finetuning, incorporating domain-specific knowledge into the training process is essential for enhancing model performance in specialized applications. Researchers can create models that provide accurate and relevant outputs tailored to local users’ needs by integrating localized datasets that include industry-specific terminology, regulations, and user-generated content. The work by Geng et al. (2024) illustrates how localized training data can lead to significant improvements in task-specific performance, such as question answering and product recommendations^13^.

Moreover, addressing the challenges of cultural representation is vital in localization efforts. Many existing multilingual models may inadvertently perpetuate biases or misunderstandings due to their reliance on predominantly Western datasets. Researchers can ensure that LLMs can handle a wide range of user queries with cultural sensitivity by focusing on diverse sources of localized content—such as community contributions, regional publications, and industry reports.

The integration of localization techniques not only enhances model accuracy but also increases user trust and engagement. Users are more likely to rely on AI systems that effectively understand their language and cultural context. In this technologically advanced yet culturally distinct market, the successful integration of AI solutions hinges on their ability to accurately interpret and respond to local linguistic nuances, industry-specific terminology, and regionally relevant content. Consequently, AI models that demonstrate a deep understanding of Taiwanese cultural contexts and business practices are more likely to gain widespread acceptance and effectively address the unique needs of local users and enterprises.

In summary, localizing LLMs for non-English languages is essential for optimizing their performance and relevance in specific contexts. By finetuning localized datasets, incorporating domain-specific knowledge, and addressing cultural nuances, researchers can develop models that meet users’ unique needs in diverse linguistic environments. This emphasis on localization is a foundational aspect of TCNNet-9 B’s development, ensuring its effectiveness in serving Taiwan’s networking industry.

### Networking domain and AI applications

The networking domain encompasses many knowledge areas, including network infrastructure, cybersecurity, hardware configuration, and telecommunications. As technology continues to evolve, the complexity of network systems has increased, necessitating the integration of artificial intelligence (AI) to address various challenges in this field. AI applications in networking are designed to automate processes, enhance security measures, optimize performance, and provide intelligent recommendations for network management.

One of the primary applications of AI in networking is in the area of cybersecurity. With the rise of sophisticated cyber threats, organizations are increasingly turning to AI-driven solutions to enhance their security posture. Machine learning algorithms can analyze vast amounts of data in real-time to detect anomalies and potential threats, enabling proactive responses to security incidents. For instance, recent studies have demonstrated that AI systems can identify patterns indicative of cyber-attacks, such as Distributed Denial of Service (DDoS) attacks or phishing attempts, significantly reducing response times and mitigating damage^14^.

In addition to cybersecurity, AI is being utilized for network optimization. Intelligent algorithms can analyze traffic patterns and resource usage to optimize bandwidth allocation and improve overall network performance. Techniques such as reinforcement learning have been applied to dynamically adjust network configurations based on real time data, ensuring that resources are utilized efficiently. The potential for AI-driven optimization is particularly relevant in environments with fluctuating demands, such as cloud computing and Internet of Things (IoT) networks^15^.

Another significant application of AI in the networking domain is in automated network management. Traditional network management often involves manual configurations and monitoring, which can be time-consuming and prone to human error. AI-powered systems can automate routine tasks such as device configuration, performance monitoring, and fault detection. By leveraging natural language processing (NLP) capabilities, these systems can also facilitate user interactions through intelligent virtual assistants that provide troubleshooting support and configuration guidance^16^.

Furthermore, AI applications in networking extend to intelligent recommendations for hardware and software solutions. AI systems can suggest optimal configurations or products tailored to specific needs by analyzing user behavior and preferences. This capability is particularly beneficial for businesses seeking to enhance their network infrastructure without extensive technical expertise.

Integrating AI into networking improves operational efficiency and enables organizations to make data-driven decisions that enhance overall performance. As networks become increasingly complex and interconnected, the demand for intelligent solutions that adapt to changing conditions will continue growing.

In summary, the networking domain presents numerous opportunities for AI applications that address critical challenges such as cybersecurity, network optimization, automated management, and intelligent recommendations. By harnessing the power of AI, organizations can improve their operational capabilities and respond more effectively to emerging threats and demands. This context underscores the relevance of TCNNet-9B as a specialized language model designed to meet the unique needs of the Taiwanese networking industry by providing accurate information and practical recommendations tailored to local users.

### Challenges and strategies in data collection for LLMs and the role of localized datasets

Effective data collection is pivotal for training high-performing large language models (LLMs). Training data’s quality, diversity, and relevance directly influence model performance, particularly when adapting LLMs for specialized applications in specific domains, such as networking and cybersecurity. Strategies such as web scraping targeted content, data augmentation, and incorporating domain-specific knowledge bases have proven effective in overcoming these challenges^17^. Ensuring data quality is particularly crucial^18^ when training models for specialized applications like TCNNet-9B. This section discusses the challenges associated with data collection and highlights the importance of localized datasets in enhancing model performance.

One of the primary challenges in data collection is ensuring the dataset’s representativeness. For models like TCNNet-9B, designed to serve the Taiwanese networking industry, gathering data that accurately reflects local terminology, cultural nuances, and specific industry practices is essential. When LLMs are trained predominantly on generalized corpora, they may struggle to understand context-specific language or provide relevant recommendations.

Another critical concern is addressing biases in training data. General-purpose datasets may inadvertently propagate stereotypes or inaccuracies related to local contexts. Researchers can mitigate these risks by utilizing localized datasets that focus on culturally relevant content and ensure that models deliver accurate and sensitive outputs to local users’ needs.

Curating localized datasets involves collecting information from various sources, including industry reports, technical documentation, user manuals, and community-generated content. This comprehensive approach allows for the inclusion of diverse perspectives and knowledge that enrich the training data. Research by Kuo (2024) emphasizes the importance of empowering communities to collaboratively curate AI evaluation datasets through systems like Wikibench. These systems capture community consensus, disagreement, and uncertainty while allowing users to shape the overall data curation process. This community-driven approach ensures that models reflect real-world usage patterns and user needs^19^.

Moreover, localized datasets significantly enhance the model’s ability to perform specific tasks effectively. For example, TCNNet-9B was trained on a dataset that included DIY assembly guides and local cybersecurity regulations, enabling it to provide practical recommendations tailored to Taiwanese users’ needs. This targeted approach improves the relevance of model outputs and increases user trust and satisfaction.

In summary, overcoming data collection challenges is essential for developing effective domain-specific LLMs. Researchers can create models that deliver accurate, contextually appropriate responses by focusing on localized datasets that capture cultural nuances and address local demands. This emphasis on quality data collection is central to the development of TCNNet-9B, ensuring its effectiveness in meeting the unique demands of Taiwan’s networking industry.

### Benchmarks and evaluation frameworks for domain-specific LLMs

Evaluating the performance of large language models (LLMs) is critical for understanding their effectiveness in specific applications, particularly in specialized domains such as networking and cybersecurity. A robust evaluation framework assesses the accuracy and relevance of model outputs and provides insights into areas for improvement, guiding further development and finetuning efforts.

Traditional evaluation metrics, such as accuracy, precision, recall, and F1-score, have been widely used to assess model performance across various tasks. However, these metrics may not fully capture language understanding and generation complexities in domain-specific contexts. For instance, the nuances of technical language and the importance of contextual relevance necessitate tailored evaluation approaches that reflect the unique requirements of specialized applications^20,21^.

Several benchmarks have been established to facilitate the evaluation of LLMs in various contexts:

- **Perplexity**: This metric assesses how well a probability model predicts a sample. In the context of language models, lower perplexity indicates better performance in predicting the next word in a sequence^16^.
- **BLEU**: Similar to ROUGE, BLEU is used to evaluate machine translation quality by comparing generated translations against reference translations. It focuses on precision while considering n-grams as well^16^.
- **ROUGE**: This metric evaluates the quality of summaries generated by models by comparing them to reference summaries. It measures recall and precision based on n-grams, making it useful for tasks involving text summarization^16^.

For specific languages such as Chinese and Taiwanese:

- **CLUE** (Chinese Language Understanding Evaluation): This benchmark encompasses a range of tasks designed to assess natural language understanding in Chinese. It includes nine tasks spanning sentence classification and machine reading comprehension, providing a comprehensive framework for evaluating models on original Chinese text. CLUE has been instrumental in advancing research in Chinese NLP by offering a standardized evaluation platform^22^.
- **TTQA** (Taiwanese Trivia Question Answering): This benchmark assesses models’ abilities to answer questions relevant to Taiwanese culture and knowledge. By providing context-specific challenges, TTQA helps evaluate how well models like TCNNet-9B can understand and generate culturally appropriate responses^23^.

Recent research has highlighted the need for custom benchmarks that align with specific tasks and challenges faced in particular domains. For example, the development of the Taiwanese Networking and Cybersecurity Knowledge Benchmark (TNCK-Bench) represents a significant advancement in evaluating models like TCNNet-9B. This benchmark includes a variety of tasks designed to assess the model’s ability to provide accurate information, answer domain-specific questions, and make relevant product recommendations based on localized knowledge.

In addition to quantitative metrics, qualitative evaluations play a crucial role in assessing LLM performance. Human evaluations can provide valuable insights into how well a model meets user expectations and addresses real-world needs. Wang et al. (2024) propose a user-centric framework that categorizes user intents and employs intent-aware assessment methods, emphasizing the importance of moving beyond traditional metrics to incorporate authentic user needs. Their research demonstrates how this approach provides more meaningful performance evaluation and suggests that properly aligned LLMs can enhance human productivity and creative potential by effectively responding to complex, multi-intent queries^24^.

Furthermore, recent advancements in interpretability techniques have enabled researchers to gain deeper insights into model behavior. Understanding how models arrive at specific outputs can inform both evaluation and future development efforts. Freedman et al. (2025) develop Argumentative Large Language Models (ArgLLMs) that integrate formal argumentation structures with LLMs, enabling transparent decision-making through contestable outputs and logical justification chains. These models achieve comparable accuracy to state-of-the-art methods while providing explainable outputs crucial for high-stakes domains like healthcare and legal decision-making, revealing potential biases and areas where models may struggle^25^.

The integration of these varied evaluation approaches is essential for developing effective domain-specific LLMs. Researchers can ensure that models like TCNNet-9B perform well in controlled settings and deliver practical value in real-world applications by employing both quantitative benchmarks and qualitative assessments.

In summary, establishing comprehensive evaluation frameworks is vital for assessing the performance of domain-specific LLMs. Custom benchmarks tailored to specific tasks—such as CLUE and TTQA—along with qualitative evaluations and interpretability techniques, provide a holistic view of model effectiveness. This focus on rigorous evaluation is central to ensuring that TCNNet-9B meets the unique needs of Taiwan’s networking industry.

### Advances in finetuning techniques for LLMs

Finetuning techniques are essential for adapting large language models (LLMs) to specific tasks and domains, allowing them to achieve higher accuracy and relevance in their outputs. As LLMs have evolved, researchers have developed various finetuning methodologies that leverage existing pre-trained models while enhancing their performance through targeted adjustments.

One of the prominent techniques in this area is transfer learning, which allows models to retain the knowledge gained from large, diverse datasets while adapting to specialized tasks. This approach has significantly reduced the labeled data required for practical training in specific domains. For example, Kamath et al. (2019) explain domain adaptation as a form of transfer learning in which the task remains the same, but the distribution changes between source and target domains. They illustrate how sentiment analysis models trained on electronic product reviews may misinterpret domain-specific phrases when applied to hotel reviews, highlighting the challenges of domain shift without extensive retraining^26^.

Another notable advancement is instruction tuning, which involves training models on datasets that include explicit instructions or prompts related to the desired tasks. This technique has enhanced the model’s ability to follow user queries and generate contextually appropriate responses. Zhao et al. (2023) propose a Self-Guide. This multi-stage mechanism synthesizes task-specific input-output pairs from the language model for self-finetuning. Their approach demonstrates substantial improvements in classification and generation tasks, enabling models to understand user intent better and provide relevant information without requiring external learning signals^27^.

Moreover, few-shot learning has emerged as a powerful strategy for finetuning LLMs with limited labeled data. This method allows models to generalize from a small number of examples, making them more adaptable to new tasks without extensive retraining. Recent studies, such as those by Perez et al. (2023) examine "true few-shot learning" where no held-out examples are available for model tuning. They reveal challenges in few-shot model selection that suggest previous studies may have overestimated the effectiveness of this approach^28^.

These finetuning techniques were integral to the development of TCNNet-9B. The model underwent continued pretraining on a carefully curated dataset, including networking knowledge, DIY assembly guides, and local cybersecurity regulations. By employing a combination of transfer learning and instruction tuning, TCNNet-9B was specifically tailored to address the unique needs of the Taiwanese networking industry.

Furthermore, researchers have begun exploring adaptive finetuning, which adjusts learning rates based on the complexity of the task or the characteristics of the dataset being used. This dynamic approach allows for more efficient training processes and can lead to better model performance across diverse applications. The findings by Lu et al. (2024) investigate various finetuning strategies, including Continued Pretraining, Supervised Finetuning, and preference-based optimization methods for domain adaptation. Their research reveals that merging multiple finetuned models can lead to emergent capabilities exceeding those of individual models, demonstrating improved performance in specialized contexts^29^.

In summary, advances in finetuning techniques play a crucial role in optimizing LLMs for specific applications. By leveraging transfer learning, instruction tuning, few-shot learning, and adaptive finetuning methods, researchers can create models that retain their general capabilities and excel in specialized domains like networking and cybersecurity. This focus on targeted adaptations is central to the development of TCNNet-9B, ensuring its relevance and effectiveness within Taiwan’s unique technological landscape.

The development of TCNNet-9B builds upon extensive research on LLMs, domain adaptation, and localization. This model aims to bridge the gap in localized, domain-specific knowledge within the networking sector by leveraging specialized datasets and advanced finetuning techniques. The following sections elaborate on this research’s methodology, results, and implications, contributing to the broader discourse on applying LLMs in specialized and non-English contexts.

## Methods

The methodology for developing TCNNet-9B involves a multistep process of data collection, pretraining, instruction finetuning, and evaluation. This section outlines each step in detail, explaining the rationale and procedures employed to create a highly specialized Traditional Chinese language model for the networking domain.

### Data collection

#### Corpus compilation

To ensure that the model has comprehensive and relevant knowledge, we compiled a rich corpus of Traditional Chinese content called TCNNet-Pretrain-NetCom-zhTW-3.7M^30^ through web scraping, including articles on networking knowledge, DIY assembly guides, equipment recommendations, and cybersecurity information pertinent to Taiwan. Additionally, local cybersecurity regulations, industry trends, and brand information were incorporated to enhance the model’s contextual understanding. Table 1 summarizes the sources and types of data collected.

**Table 1:** Summary of data sources.

Data source type	Description	Websites	# of raw documents	# of preprocessed documents	Total token count
Networking knowledge	Articles and technical documents on networking concepts, protocols, and best practices from reputable sources.	msi.com asus.com dnpz.net	1609	1532	1.421 millions
DIY assembly guides	Detailed tutorials and guides for assembling networking equipment.	kocpc.com.tw kaiching.org tangpc.com.tw msi.com dnpz.net	163	161	0.139 millions
Equipment recommendations	Reviews and recommendations for various networking devices tailored to different budgets and requirements.	cool3c.com tech-girlz.com myfeel-tw.com techbang.com onnidaily.com ching3c.com ahui3c.com eprice.com.tw	2178	1963	2.019 millions
Cybersecurity Information	Articles on cybersecurity measures, threats, and best practices relevant to the Taiwanese context.	gss.com.tw ithome.com.tw iektrends.iek.org.tw	1201	1120	1.035 millions
Local regulations and industry trends	Documents on Taiwanese cybersecurity regulations, local network equipment brands, and industry development trends.	gss.com.tw acw.org.tw	77	76	0.154 millions

#### Data processing and cleaning

The collected data underwent rigorous cleaning and preprocessing to ensure quality and consistency. The quantitative data for the preprocessing is also include in Table 1. The steps included:

- Reduce content duplication.

To address the challenge of content duplication, a sophisticated hybrid approach was implemented, leveraging two complementary techniques. Initially, SimHash was utilized for rapid clustering, providing a computationally efficient method to identify potential duplicate groups. This preliminary step was followed by applying **FAISS** (Facebook AI Similarity Search), a state-of-the-art library designed for efficient large-scale similarity search. The integration of these methods enabled a robust and scalable solution for managing content similarity across an extensive corpus of articles, balancing computational efficiency with high-precision similarity detection. This process effectively handled thousands of articles with significantly reduced computational overhead.

The deduplication workflow consisted of:

1. Initial clustering using SimHash fingerprints to identify potential duplicate groups quickly.
2. High-performance similarity search using FAISS.
  Converting articles to dense vectors using a multilingual embedding model.
  Building a FAISS index for efficient nearest neighbor search.
  Performing batch similarity queries with a 90% similarity threshold.
3. Merging of similar content groups based on the FAISS similarity results.

- Content quality filtering.

1. Content length validation to filter out extremely short articles or abnormally long content that might indicate crawling errors.
2. Text preprocessing to remove HTML artifacts and standardize punctuation.
3. Knowledge density evaluation, removing articles with excessive promotional content or irrelevant personal anecdotes.

This optimized approach ensured both efficiency in processing large-scale data and quality in the final dataset, maintaining unique, high-quality technical content suitable for training purposes.

### Continuous pretraining

The continuous pretraining phase was designed to augment the base model’s comprehension of Traditional Chinese networking content. This process aimed to significantly enhance the model’s capacity for generating contextually appropriate and precise responses within the specialized domain. The pretraining regimen sought to refine the model’s linguistic understanding and domain-specific knowledge by exposing it to a curated corpus of relevant materials, improving its overall performance in tasks related to Traditional Chinese networking terminology and concepts.

#### Base model selection

We evaluated multiple candidate models to determine the most suitable base model by conducting preliminary continuous pretraining using approximately 1% of the total dataset. Each model’s performance was assessed utilizing perplexity metrics. The model with the lowest perplexity was selected as the final base model for further pretraining. This rigorous selection process ensured that the chosen base model, Yi-1.5-9B, had the best foundational performance for our domain-specific enhancements.

#### Pretraining procedure

The pretraining was onducted via a distributed training setup, leveraging multiple GPUs to handle the computational demands. The key steps included the following:

- **Initialization**: Load the base model and prepare the pretraining environment.
- **Data feeding**: The cleaned and preprocessed dataset is sequentially fed into the model.
- **Gradient accumulation**: Accumulating gradients to optimize the model’s parameters efficiently.
- **Checkpointing**: Periodically saving model checkpoints to ensure training progress and enable recovery from potential interruptions.
- **Evaluation**: Regularly evaluating the model’s performance on a validation set to monitor improvements and adjust training parameters accordingly.

##### System specifications

The pretraining process was carried out on a high-performance computing infrastructure to handle the computational demands efficiently. The system specifications are summarized in Table 2.

**Table 2:** System specifications.

Component	Specification
GPU	NVIDIA H100, 80 GB VRAM
Number of GPUs	2
CPU	Intel(R) Xeon(R) Gold 6426Y, 2.50 GHz
RAM	259 GB
Storage	1.8 TB NVMe SSD
Framework	PyTorch
Distributed training	NCCL (NVIDIA Collective Communication Library)
Training time	Approximately 2 weeks

#### Performance monitoring

Throughout the pretraining process, various metrics were tracked to ensure that the model’s performance aligned with our objectives:

- **Perplexity**: Measuring the model’s ability to predict the next token in a sequence, with lower values indicating better performance.
- **Token coverage**: Ensuring the model’s vocabulary coverage was comprehensive and relevant to the networking domain.

This detailed approach ensured that TCNNet-9B was robustly pre-trained on domain-specific content, significantly enhancing its ability to provide accurate and contextually relevant responses in the networking domain.

### Instruction finetuning

**Fig. 1:**
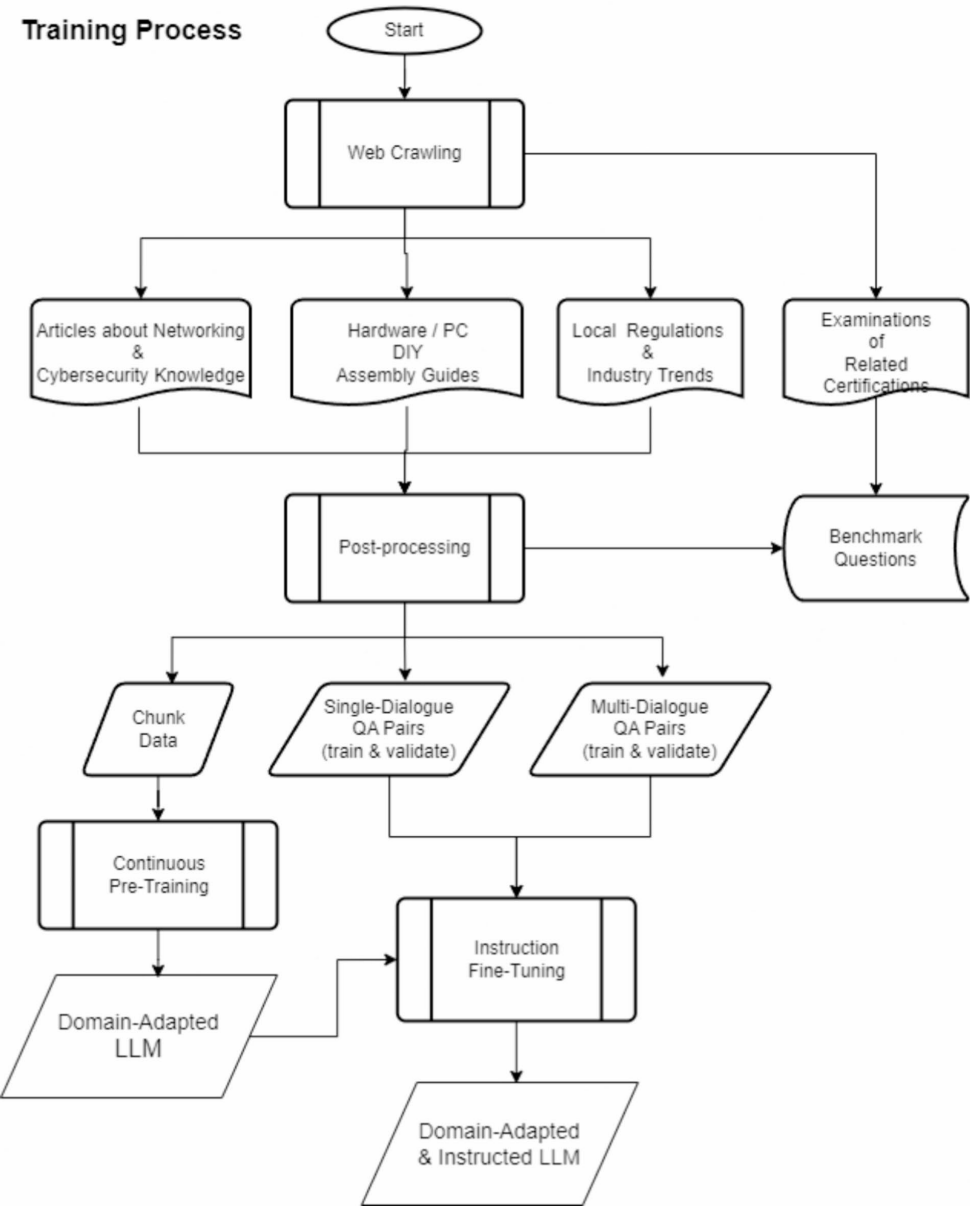
TCNNet-9B training process.

Finetuning the pre-trained TCNNet-9B model involved refining its responses to better handle specific tasks within the networking domain. This phase was crucial for adapting the model to practical applications, ensuring it could effectively address user queries and provide relevant product recommendations. The finetuning dataset comprising 1.1 million instruction-following samples in Traditional Chinese, focusing on networking and communication technology concepts, conducted with generated Q&A pairs from crawled articles and conversations from Hi5 GraphRAG chat service is also publicly accessible through the Hugging Face Datasets repository^31^. The whole finetuning process is shown in Fig. 1, which used two primary processed training datasets: Q&A and product recommendations.

#### Q&A finetuning

##### Dataset creation

For Q&A finetuning, we needed a dataset that accurately represented the types of questions users might ask in the networking domain. The process entailed systematically transforming the pretraining corpus into a structured question-and-answer (Q&A) format. This critical step involved leveraging advanced natural language processing techniques to extract relevant information from the corpus and reformulate it into coherent question-answer pairs. The structured Q&A format was designed to simulate real-world queries and responses, enhancing the model’s ability to comprehend and generate contextually appropriate answers within the networking domain. Using GPT-4o, we generated both single-turn and multiturn Q&A pairs. Single-turn Q&A pairs addressed straightforward questions, whereas multiturn Q&A pairs simulated more complex interactions requiring follow-up questions and contextual understanding. The questions covered various topics, including networking protocols, equipment troubleshooting, DIY assembly steps, and cybersecurity practices.

##### Finetuning process

The finetuning process for Q&A involves creating a dataset that accurately represents user questions in the networking domain. We utilized LLM Prompt Engineering to extract article knowledge into single-turn and multi-turn Q&A pairs; the prompts and generated Q&A pair examples are shown in Tables 3 and 4 from the pretraining corpus via GPT-4o, ensuring diverse and contextually relevant queries. The generation of Q&A pairs training data prioritized accuracy, so we applied strict parameter settings with a temperature of 0 and a repetition penalty of 1. The model was finetuned through data augmentation, specialized training configuration, and iterative validation. Data augmentation included paraphrasing and varying question formats, while the training configuration optimized hyperparameters such as the learning rate and batch size. Continuous validation against a separate dataset allowed adjustments to improve response accuracy and contextual relevance.

**Table 3:**
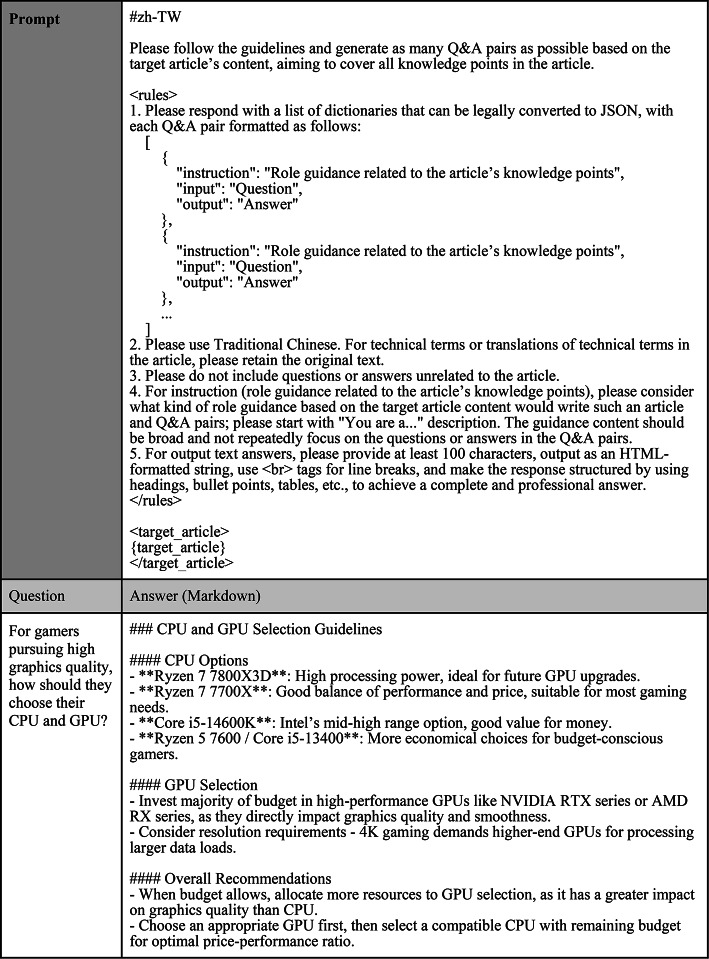
Prompt and examples of generated single-turn Q&A pairs for finetuning.

**Table 4:**
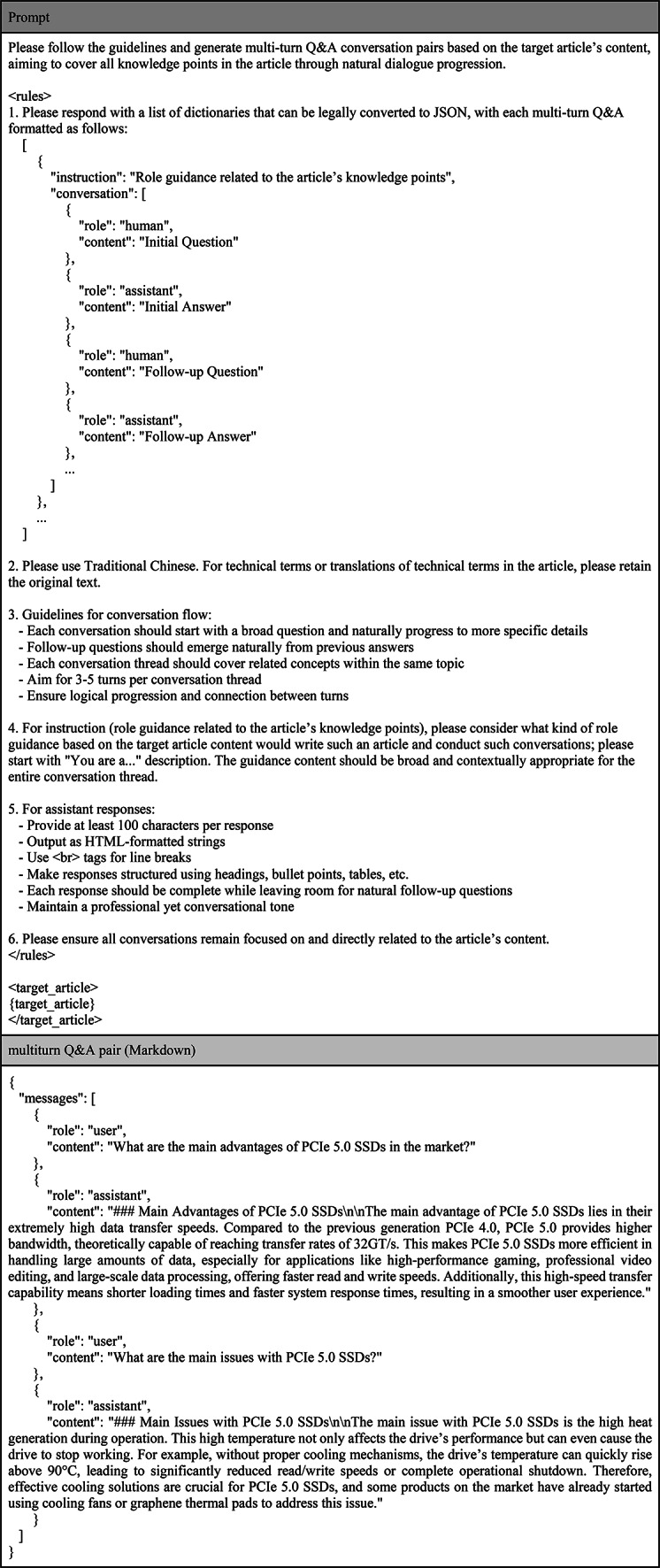
Examples of multi-turn Q&A pairs for finetuning.

#### Product recommendation finetuning

##### Dataset source

We utilized outputs from Hi5’s intelligent sales advisor, which integrates GraphRAG technology, to finetune product recommendations. This system provides a wealth of single-turn and multi-turn product recommendation and comparison datasets. These datasets included scenarios where users sought advice on selecting networking equipment, comparing products based on specific criteria, and receiving tailored recommendations based on their needs and budget.

##### Finetuning process

For product recommendation finetuning datasets, as shown in Tables 5 and 6, we utilized the real conversation outputs from Hi5’s intelligent sales advisor. The training focused on understanding the user context, generating coherent and informative responses, and performing comparative analysis. The model was trained to recognize keywords indicating user intent, generate detailed and relevant product recommendations, and maintain consistency in multi-turn interactions. This approach ensured that TCNNet-9B could provide personalized and accurate product suggestions tailored to user needs and preferences.

**Table 5:** Examples of single-turn product recommendation for finetuning.

Question	Answer (Markdown)
Looking for a smart switch with built-in power management system?	1. MikroTik Switch CSS326-24G-2 S + RM - Recommended because: This switch features a built-in power management system and comes with 24 Gigabit Ethernet ports plus 2 SFP + ports, offering line-rate connectivity. It supports various switching functions, including port-to-port forwarding management, MAC filtering, VLAN configuration, traffic mirroring, and bandwidth limiting capabilities. It’s particularly suitable for network environments requiring high performance and flexible management.

**Table 6:**
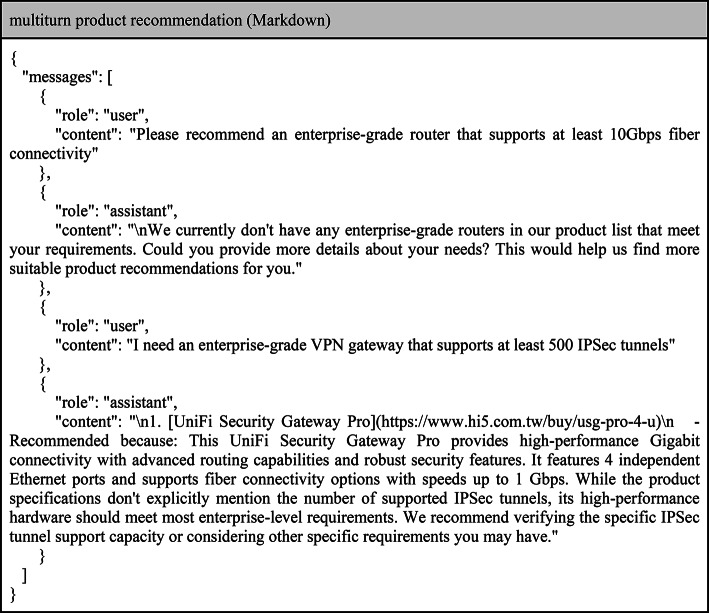
Examples of multiple-turn product recommendations for finetuning.

Table 7 notes the finetuning configurations. This comprehensive approach to instruction finetuning ensured that TCNNet-9B was well-equipped to handle the diverse and complex queries typical of the networking domain, providing accurate, relevant, and contextually appropriate responses to users.

**Table 7:** Configurations of instruction finetuning.

Parameters	Value
Epochs	10
Batch size (per device)	1
Gradient accumulation steps	4
Learning rate	5e-5
Warm-up ratio	0.03
Save steps	100
LR scheduler type	cosine
‍Finetuning algorithm	LoRA

### Benchmark dataset creation

To evaluate TCNNet-9B’s understanding of the Taiwanese networking and cybersecurity domain, we developed a specialized benchmark named the “**Taiwanese Networking and Cybersecurity Knowledge Benchmark (TNCK-Bench)**.” This benchmark assessed the model’s proficiency in handling domain-specific knowledge pertinent to the Taiwanese context. The TNCK-Bench consists of two main components:

- **Certification Test Questions**: Multiple-choice questions sourced from certification tests of international networking equipment brands, such as the MikroTik Certified Network Associate (MTCNA) Examination. These questions evaluated the model’s understanding of standard networking protocols, equipment features, and best practices recognized by leading industry certifications. We translated historical examination questions from English to Traditional Chinese for the Taiwanese version benchmark.
- **Localized Knowledge Questions**: These are multiple-choice questions derived from the Taiwan National Information Security Higher Examinations. This component aims to test the model’s grasp of Taiwanese local cybersecurity standards and practices.

### Comparative evaluation

To comprehensively evaluate the performance of TCNNet-9B, we conducted a comparative analysis against several baseline models. This section details the metrics used for evaluation and the results obtained. The evaluation of TCNNet-9B involves multiple metrics to ensure a thorough assessment of its capabilities:

- **Benchmark score results**: The primary metric was the accuracy score on the TNCK-Bench, which represents the percentage of correctly answered multiple-choice questions. This score directly reflects the model’s proficiency in the networking and cybersecurity domain, which is specific to Taiwan.
- **Response similarity**: We assessed the similarity between the model’s responses and the correct answers in the test set. This metric calculates the cosine similarity between the model’s generated responses and the ground truth answers, providing a quantitative measure of how closely the model’s outputs match the expected responses.
- **Custom quality evaluation Criteria**: To evaluate the quality of the model’s responses in practical applications, we defined several custom criteria tailored to the networking domain and product recommendation tasks. These criteria included the following.
  1. **Technical accuracy**: This metric measures the correctness of the model’s technical information. Higher scores indicate that the model’s responses accurately reflect networking concepts, protocols, and best practices. TCNNet-9B demonstrated superior technical accuracy compared to the baseline model, indicating enhanced understanding and application of domain-specific knowledge.
  2. **Relevance**: This metric evaluates how well the model’s responses align with the user’s query. Higher scores suggest that the responses are directly relate to the questions asked and appropriately address the user’s needs. TCNNet-9B achieved higher relevance scores, reflecting its improved ability to generate contextually appropriate and helpful responses.
  3. **Details and depth**: This metric assesses the comprehensiveness of the model’s responses, including the level of detail and depth provided. Higher scores indicate that the model’s answers are thorough and informative. TCNNet-9B received higher scores for detail and depth, demonstrating its ability to provide more elaborate and in-depth explanations and recommendations.
  4. **Clarity and Coherence**: This metric measures the clarity and logical consistency of the model’s responses. Higher scores indicate that the responses are easy to understand and logically structured. TCNNet-9B showed improved clarity and coherence, ensuring its responses were intelligible and well organized.

## Results

This section presents the results of the continued pretraining, instruction finetuning, and benchmarking of TCNNet-9B. The model’s performance was evaluated through comprehensive experiments and comparisons with baseline models.

### Pretraining results

We initially evaluated multiple candidate models to identify the most suitable base model for continued pretraining. Each model was pre-trained on approximately 1% of the total dataset, and its performance was assessed using perplexity metrics. The results of these preliminary experiments are summarized in Table 8.

**Table 8:** Continuous pretraining performance (1% of the total dataset) of the base models.

Base model	Perplexity
Meta-Llama-3-8B	7.270
Meta-Llama-3-8B-Instruct	10.948
Llama3-TAIDE-LX-8B-Chat-Alpha1	18.369
Yi-1.5-9B	**5.685**
Yi-1.5-9B-Chat	6.287
Breeze-7B-Instruct-v1_0	7.742
Qwen2-7B	7.768
Qwen2-7B-Instruct	8.570
glm-4-9b	6.411
glm-4-9b-chat	7.154

Based on these evaluations, Yi-1.5-9B demonstrated the lowest perplexity and highest accuracy, making it the optimal choice for our base model. We then proceed with the continued pretraining phase using Yi-1.5-9B, which significantly enhances TCNNet-9B’s understanding of Traditional Chinese networking content. The performance improvements from the continued pretraining phase are detailed below in Table 9.

**Table 9:** Continuous pretraining performance (full dataset) on Yi-1.5-9B.

Base model	Perplexity
Yi-1.5-9B	**3.724**

The lower perplexity and higher accuracy on the pretraining data indicate that TCNNet-9B has successfully internalized domain-specific knowledge and can generate relevant responses. These results validate the effectiveness of our approach in selecting and further pretraining the base model to meet the specific requirements of the Taiwanese networking domain.

### Q&A finetuning results

Finetuning TCNNet-9B on the Q&A datasets significantly improved its performance in generating accurate and contextually appropriate answers, as shown in Table 10. We used the ROUGE-L and BLEU-4 metrics to measure the similarity between the finetuned model’s responses and the ground truth answers in the test set. These metrics provide a robust evaluation of the model’s ability to produce relevant and precise responses.

**Table 10:** Q&A task finetuning performance comparison (*-cpt represents the continuous pretrained model).

Models	Before finetuning		After finetuning	
	BLEU-4	ROUGE-L	BLEU-4	ROUGE-L
Meta-Llama-3-8B	4.983	10.903	5.210	11.112
Meta-Llama-3-8B-cpt	4.356	10.386	6.021	12.042
Meta-Llama-3-8B-Instruct	3.513	8.594	3.509	8.757
Meta-Llama-3-8B-Instruct-cpt	3.467	8.787	3.524	8.757
Yi-1.5-9B	6.943	13.082	7.532	13.534
Yi-1.5-9B-cpt	7.075	13.135	**27.599**	**31.834**
Qwen2-7B	12.694	20.122	14.774	23.491
Qwen2-7B-cpt	12.110	19.433	13.473	22.524
Qwen2-7B-Instruct	14.999	23.161	24.504	31.397
Qwen2-7B-Instruct-cpt	15.508	23.688	15.508	23.688
glm-4-9b	7.896	13.848	22.633	27.656
glm-4-9b-cpt	7.701	13.883	24.162	29.472
glm-4-9b-chat	9.565	16.571	25.382	30.021
glm-4-9b-chat-cpt	9.398	16.222	25.972	30.145

The higher ROUGE-L and BLEU-4 scores suggest that the finetuned model produces responses more similar to the ground-truth answers regarding length and word choice. This enhanced similarity demonstrates that finetuning Yi-1.5-9B-cpt (TCNNet-9B) improved the ability to generate contextually relevant and accurate responses, making it better suited for handling domain-specific queries in the networking field.

### Product recommendation finetuning results

The product recommendation finetuning phase enhanced TCNNet-9B’s ability to deliver personalized and accurate product suggestions. The effectiveness of this finetuning was evaluated via three key metrics from the RAGAS framework: context relevancy, consistency with the given context, and answer relevancy. In Table 11, these metrics help assess whether the finetuned model provides more precise and contextually appropriate recommendations than the baseline model.

**Table 11:** Product recommendation task finetuning performance comparison.

Models	Yi-1.5-9B	TCNNet-9B
Context relevancy	0.043975	0.167531
Consistency with given context	0.426159	0.651889
Answer relevancy	0.372635	0.482500

- **Context relevancy**: This metric measures how well the model’s recommendations align with the context provided by the user query. A higher score indicates that the model effectively understands the user’s requirements and situational details. TCNNet-9B showed significant improvements in context relevancy, reflecting its enhanced ability to grasp the nuances of user queries and provide appropriate recommendations.
- **Consistency with the given context**: This metric evaluates the coherence and logical consistency of the model’s responses concerning the given context. Higher scores indicate that the model’s recommendations are relevant and consistent with the user’s stated needs and preferences. TCNNet-9B demonstrated a higher consistency score, indicating that its recommendations were more logically aligned with the provided context than the baseline model was.
- **Answer Relevancy**: This metric assesses the overall relevance of the model’s recommendations to the user’s query. A higher score suggests that the model is better at selecting and suggesting products that meet the user’s criteria and preferences. TCNNet-9B achieved a higher answer relevancy score, highlighting its improved ability to generate relevant product suggestions that closely match user requirements.

The finetuned TCNNet-9B outperforms the baseline model across all three RAGAS metrics, demonstrating its enhanced proficiency in delivering accurate, contextually relevant, and consistent product recommendations. This improvement underscores the effectiveness of the finetuning process in refining the model’s ability to meet user needs in the networking domain.

### Benchmark results

TCNNet-9B was benchmarked against other models via the Taiwanese Networking and Cybersecurity Knowledge Benchmark (TNCK-Bench) and the English version benchmark (NCK-Bench). This benchmark evaluates explicitly the model’s understanding and application of knowledge within the Taiwanese networking and cybersecurity domain, as shown in Tables 12 and 13. The TNCK-Bench comprises a comprehensive set of multiple-choice questions meticulously curated from two primary sources: internationally recognized industry-standard networking equipment certification examinations and locally relevant networking and cybersecurity publications. This dual-source approach ensures that the benchmark effectively assesses global industry standards and region-specific knowledge, providing a robust evaluation framework for model performance in the Taiwanese networking and cybersecurity domain. The score is calculated as the percentage of correctly answered questions, providing a straightforward measure of each model’s proficiency.

**Table 12:** TNCK-Bench score for model comparison.

Models	Benchmark score
gpt-4o	67.7939
gpt-3.5-turbo	66.3969
TCNNet-9B	**53.4782**
gemini-1.5-pro	50.0611
gemini-1.0-pro	44.2137
Yi-1.5-9B	38.8554

**Table 13:** NCK-Bench score on models’ comparison.

Models	Benchmark score
gpt-4o	71.9322
gpt-3.5-turbo	68.1923
TCNNet-9B	**55.8192**
gemini-1.5-pro	50.1721
gemini-1.0-pro	45.0921
Yi-1.5-9B	40.1330

The results demonstrate that TCNNet-9B outperforms the baseline models, achieving a higher benchmark score. This finding indicates that TCNNet-9B has a superior understanding of Traditional Chinese networking and cybersecurity content, making it better suited for applications in the Taiwanese context.

Using TNCK-Bench as the evaluation metric, we ensured that the assessment focused on domain-specific knowledge critical to our target audience. The high benchmark score of TCNNet-9B underscores the effectiveness of our continued pretraining and finetuning processes, validating the model’s ability to provide accurate and contextually relevant information in the networking domain.

### Custom quality evaluation results

To further evaluate the quality of TCNNet-9B’s responses, we used custom quality evaluation metrics tailored for the networking domain and product recommendation tasks. First, we asked networking experts to design evaluation metrics for LLM response quality, ultimately determining four criteria: technical accuracy, relevance, detail and depth, and clarity and coherence. Each metric was scored on a scale from 1 to 5, with higher scores indicating better performance. Then, through prompt engineering, we designed scoring prompts for each aspect. Using a different LLM than the one used during inference, we scored each evaluation aspect of the responses, ultimately producing the score table shown in Table 14.

**Table 14:** Custom quality evaluation results comparison.

Base model	Technical accuracy	Relevance	Detail and depth	Clarity and coherence
gpt-4o	3.871	3.912	4.121	4.324
TCNNet-9B	3.631	3.839	4.087	4.110
gemini-1.5-pro	3.214	3.452	4.021	4.016
Yi-1.5-9B	2.845	2.820	3.423	3.786

The custom quality evaluation results highlight the significant improvements achieved by TCNNet-9B over the baseline model. These enhancements underscore the effectiveness of our finetuning approach in refining the model’s ability to deliver high-quality, domain-specific responses in the context of networking and product recommendation.

The results show that TCNNet-9B’s performance in terms of these metrics is comparable to and, in some cases, close to that of GPT-4o. This finding demonstrates that smaller or medium-sized models can improve performance levels on specific tasks when subjected to continued pretraining and finetuning. This finding validates the effectiveness of specialized training methods in enhancing the capabilities of smaller models, making them viable alternatives for domain-specific applications.

## Conclusion

This paper presented TCNNet-9B, a specialized Traditional Chinese language model tailored for the networking domain, built upon the open-source Yi-1.5-9B model. Through continued pretraining and finetuning, TCNNet-9B was developed to meet the unique needs of the Taiwanese networking industry, addressing a gap in localized and domain-specific knowledge.

### Key findings

The experimental results demonstrated significant improvements across multiple performance metrics.

- **Benchmark performance**: TCNNet-9B outperforms baseline models in the Taiwanese Networking and Cybersecurity Knowledge Benchmark (TNCK-Bench), demonstrating superior understanding and application of domain-specific knowledge.
- **Q&A task performance**: In the Q&A tasks, TCNNet-9B showed enhanced performance, as measured by the ROUGE-L and BLEU-4 metrics, indicating improved accuracy and relevance in generating responses.
- **Product recommendation task performance**: For product recommendation tasks, the model achieved higher scores in the RAGAS framework metrics: Context Relevancy, Consistency with the Given Context, and Answer Relevancy, reflecting better precision and contextual appropriateness.

### Contributions to the field

This research contributes to the field of NLP and LLMs in several ways:

- **Domain-specific adaptation**: This demonstrates the value of domain-specific adaptation through continued pretraining on specialized corpora, which significantly enhances model performance.
- **Localization for non-english contexts**: This highlights the potential for developing high-performance models in languages other than English that cater to the specific needs of different linguistic and cultural contexts.

### Future work

Several areas for future research and development have been identified to enhance TCNNet-9B further:

- **Iterative finetuning**: Enhancing the accuracy and relevance of TCNNet-9B through continuous retraining with updated datasets and parameter adjustments based on user feedback.
- **System enhancements**: Optimize the API infrastructure and user interface to improve data flow, reduce response latency, and enhance the user experience.
- **Feature expansion**: Develop advanced recommendation algorithms, incorporate new data sources for enriched responses, and enhance the model’s ability to handle emerging networking trends and technologies.

The development of TCNNet-9B represents a significant step forward in the specialization and localization of large language models. By addressing the specific needs of the Taiwanese networking industry and demonstrating the effectiveness of domain-specific and culturally relevant training, this research lays the groundwork for future advancements in the field. As the landscape of AI and NLP continues to evolve, the insights gained from this work will contribute to the ongoing quest for more intelligent, adaptive, and context-aware language models.

## Data Availability

The datasets generated and/or analyzed during the current study are publicly available in the following repositories on the Hugging Face platform:1. TCNNet-Pretrain-NetCom-zhTW-3.7 M dataset: https://huggingface.co/datasets/DataAgent/TCNNet-Pretrain-NetCom-zhTW-3.7M2. TCNNet-SFT-NetCom-zhTW-1.1 M dataset: https://huggingface.co/datasets/DataAgent/TCNNet-SFT-NetCom-zhTW-1.1MThese datasets consist of articles written in Traditional Chinese in domains such as networking, cybersecurity, and tech reviews. They were utilized for the continuous pretraining of the TCNNet-9B model. The datasets have been properly curated and are publicly accessible.
